# High glucose induces an early and transient cytoprotective autophagy in retinal Müller cells

**DOI:** 10.1007/s12020-022-03079-8

**Published:** 2022-05-25

**Authors:** A. Mecchia, C. Palumbo, A. De Luca, D. Sbardella, A. Boccaccini, L. Rossi, M. Parravano, M. Varano, A. M. Caccuri

**Affiliations:** 1 IRCCS-G.B. Bietti Foundation, Rome, Italy; 2 Department of Clinical Sciences and Translational Medicine, University of Rome Tor Vergata, Rome, Italy; 3 Department of Biology, University of Rome Tor Vergata, Rome, Italy; 4 Department of Chemical Sciences and Technologies, University of Rome Tor Vergata, Rome, Italy; 5 The NAST Centre for Nanoscience and Nanotechnology and Innovative Instrumentation, University of Rome Tor Vergata, Rome, Italy

**Keywords:** Retina, Glial cells, Diabetes, Autophagy, ERK

## Abstract

**Purpose:**

We investigated the autophagic response of rat Müller rMC-1 cells during a short-term high glucose challenge.

**Methods:**

rMC-1 cells were maintained in 5 mM glucose (LG) or exposed to 25 mM glucose (HG). Western blot analysis was used to evaluate the expression levels of markers of autophagy (LC3-II, p62) and glial activation (AQP4), as well as the activation of TRAF2/JNK, ERK and AKT pathways. Autophagic flux assessment was performed using the autophagy inhibitor chloroquine. ROS levels were measured by flow cytometry using dichlorofluorescein diacetate. ERK involvement in autophagy induction was addressed using the ERK inhibitor FR180204. The effect of autophagy inhibition on cell viability was evaluated by SRB assay.

**Results:**

Activation of autophagy was observed in the first 2–6 h of HG exposure. This early autophagic response was transient, not accompanied by an increase in AQP4 or in the phospho-activation of JNK, a key mediator of cellular response to oxidative stress, and required ERK activity. Cells exposed to HG had a lower viability upon autophagy inhibition by chloroquine, as compared to those maintained in LG.

**Conclusion:**

A short-term HG challenge triggers in rMC-1 cells a process improving the ability to cope with stressful conditions, which involves ERK and an early and transient autophagy activation.

## Introduction

Retinal Müller cells are the most widespread glial cells in the retina. They are mainly found in the inner nuclear layer but are able to reach all nervous layers of the retina [1]. Under stress conditions, such as high glucose (HG), Müller cells respond through reactive gliosis. This results in morphological, biochemical and physiological changes, which favor the onset of diabetic retinopathy (DR) [2]. Therefore, it is important to understand the molecular mechanisms modulating the response to HG in different kinds of retinal cells in experimental conditions mimicking DR.

Among the consequences of exposure to a HG environment are reactive oxygen species (ROS) accumulation, and endoplasmic reticulum (ER) stress signaling pathway activation that could induce autophagy [3, 4]. This intracellular degradation system maintains cellular homeostasis by removing abnormal proteins and damaged organelles like mitochondria. Moreover, induction of autophagy has been reported to attenuate HG-induced oxidative injury in lens epithelial cells [5]. However, excessive or uncontrolled levels of autophagy are able to induce apoptosis or other forms of autophagy-dependent cell death [6, 7].

The rat retinal Müller cell line rMC-1 expresses both induced and basal markers found in primary Müller cell cultures and represents an important tool for studying the regulation of autophagy by glucose. Lopes de Faria et al. have investigated in this cell line the effect on autophagy of glucose concentrations that mimic diabetic conditions (25 mM glucose, HG), in a time interval ranging between 24 and 72 h. HG exposure increased the amount of autophagosomes but failed to degrade sequestosome 1 (p62), while treatment with the mTOR inhibitor rapamycin restored lysosomal proteolytic activity and reactivated the autophagic flux [8]. Based on these results the authors concluded that Müller cells are unable to respond adequately to glucose-induced stress, due to lysosomal impairment. Similar conclusions were drawn by Wang et al. in a study performed using primary rat Müller cells [9]. In this study, exposure to HG for 24 h caused downregulation of the autophagy-related protein Beclin-1, accumulation of p62 and autophagosomes and decrease of autolysosomes, consistent with an inhibition of autophagy. However, according to other authors, autophagy induction was observed in rMC-1 cells after 48 h of treatment with HG [10]. Further, it has been reported that in rMC-1 cells treated with HG autophagic markers, such as Beclin 1 puncta, were not seen up to 72 h following HG exposure [11] and that mitophagy was induced in rMC-1 [12] and MIO-M1 Müller cell lines [13] after 5 days of HG treatment. Based on these reports, at a first glance the effect of glucose on Müller cells autophagy appears controversial.

In the present study we show that in rMC-1 cells HG induces an early and cytoprotective autophagic response that peaks in the first 6 h of exposure, and that is followed by a later decline. Our findings, coupled with those reported above from other authors, indicate that in Müller cells HG triggers a non-linear response characterized by autophagic oscillations.

## Materials and methods

### Cell culture and treatments

The immortalized rat retinal Müller cell line (rMC-1) was obtained from Kerafast (Kerafast, Boston, MA, USA) [14], and routinely maintained at 37 °C, 5% CO_2_ in Dulbecco’s Modified Eagle Medium (Sigma-Aldrich, St. Louis, MO, USA), supplemented with 10% fetal bovine serum (Euroclone, Milan, Italy), 100 U/ml of penicillin, 100 μg/ml streptomycin (Sigma-Aldrich), and containing either 5 mM (1 g/l) glucose (LG) or 25 mM (4.5 g/l) glucose (HG). Mannitol (Sigma-Aldrich), added at a 20 mM concentration to a medium containing 5 mM glucose, was used to study the effect of osmolarity in cells exposed to HG. The ERK inhibitor FR180204 (SML0320, Sigma-Aldrich) was used at 10 μM and added 2 h before cells were harvested for western blotting analysis. Autophagic flux was evaluated in cells maintained under LG or HG conditions for 6, 24 or 72 h in the absence or presence of 20 μM chloroquine diphosphate (CQ) (C6628, Sigma-Aldrich), added 6 h before cell harvesting.

### Western blot analysis

Cells were lysed in RIPA buffer (25 mM Tris-HCl pH 7.6, 150 mM NaCl, 1% NP-40, 1% Sodium Deoxycholate, 0.1% SDS) supplemented with protease and phosphatase inhibitors (Sigma-Aldrich). Protein content was measured with the Pierce BCA Protein Assay kit (Thermo Fisher Scientific, Waltham, MA, USA). Western blot analysis was performed using the following primary antibodies: anti-TRAF2 (cat# 4712, Cell Signaling Technology, Danvers, MA, USA), anti‐phospho‐JNK (cat# 4668S, Cell Signaling Technology), anti-JNK (cat# 3708S, Cell Signaling Technology), anti-phospho-ERK1/2 (cat# 4370, Cell Signaling Technology), anti-ERK1/2 (cat# 4695, Cell Signaling Technology), anti-phospho-AKT (cat# 4060S, Cell Signaling Technology), anti-AKT (cat# 4685S, Cell Signaling Technology), anti-LC3 (cat# NB100-2220, Novus Biological, Littleton, CO, USA), anti-p62/SQSTM1 (cat# ab56416, Abcam, Cambridge, UK), anti-AQP4 (cat# sc-32739 Santa Cruz, Heidelberg, Germany), anti-β-actin (cat# A2066, Sigma-Aldrich). Signals were detected using appropriate horseradish peroxidase conjugated secondary antibodies and the enhanced chemiluminescence system ECL LiteAblot Extend (EuroClone, Milano, Italy). Chemiluminescence signals were captured with a digital imaging equipment (ImageQuant LAS 4000 mini). Densitometric analysis of bands was performed using the NIH ImageJ software (National Institutes of Health, Bethesda, MD, USA).

### ROS evaluation

Intracellular ROS levels were evaluated using dichlorofluorescein diacetate (DCFH-DA), as previously described [15]. Briefly, cells maintained in LG or HG for up to 6 h were incubated with 20 µM DCFH-DA in the dark, for 15 min at 37 °C and 5% CO_2_. Next, the cells were detached by trypsinization, washed three times with PBS and subjected to flow cytometric analysis (FACS Calibur, BD) to measure the conversion of DCFH-DA to the fluorescent product dichlorofluorescein.

### Cell viability studies

Cells were seeded, allowed to adhere overnight and then cultured in either LG or HG for 24 h, in the presence or absence of CQ (1–25 μM). Cell viability was then evaluated by the sulforhodamine B (SRB) assay, performed as previously described [16].

### Statistical analysis

Statistical analysis was performed by two‐tailed Student’s *t* tests, with a significance threshold set at *p* < 0.05, using GraphPad Prism (GraphPad Software, La Jolla, CA). All the experiments were repeated at least three times.

## Results

### HG triggers an early autophagy activation followed by a decline in rMC-1 cells

**Fig. 1:**
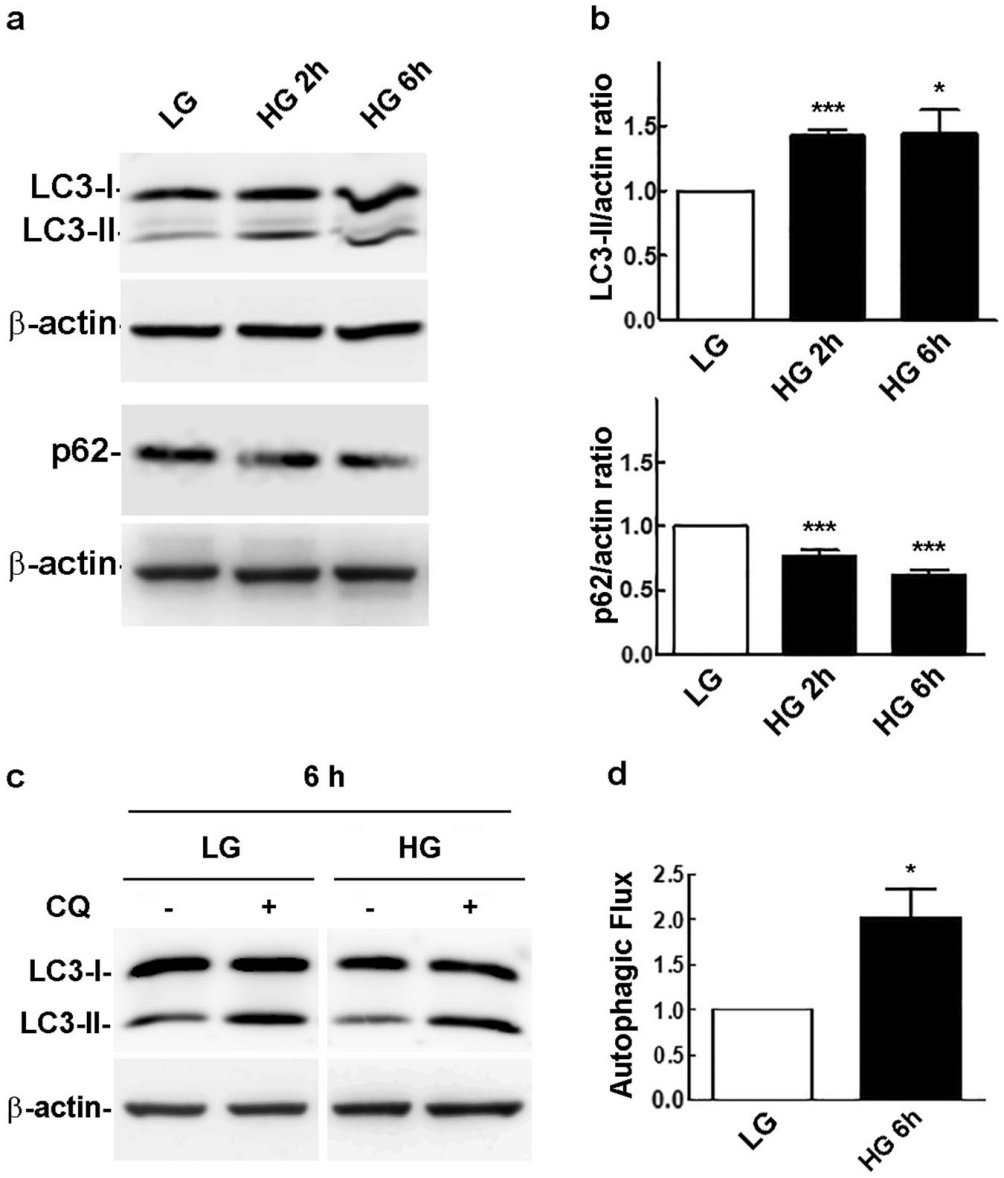
Western blot analysis of autophagy markers in cells exposed to LG or HG conditions for 2–6 h. **a** Assessment of LC3-II and p62 protein levels in cells exposed to HG for 2 and 6 h. **b** Densitometric analysis of the signals obtained in replicates of the experiments shown in **a**. LC3-II/actin and p62/actin ratios of HG-treated samples were normalized to those of the control cells maintained in LG, which were arbitrarily set equal to 1. Bars, SEM; *p < 0.05 and ***p < 0.001 vs. controls. **c** Western blot analysis of LC3-II levels, in cells maintained in either LG or HG for 6 h, in the absence or presence of CQ (20 µM). **d** Autophagic flux: LC3-II/actin ratios were determined from the densitometric analysis of the signals obtained in replicates of the experiment shown in **c**; the autophagic flux, of cells maintained in either LG or HG, was calculated as the difference between LC3-II/actin levels in the presence and absence of CQ. Bars, SEM; *p < 0.05 vs. LG-treated cells

**Fig. 2:**
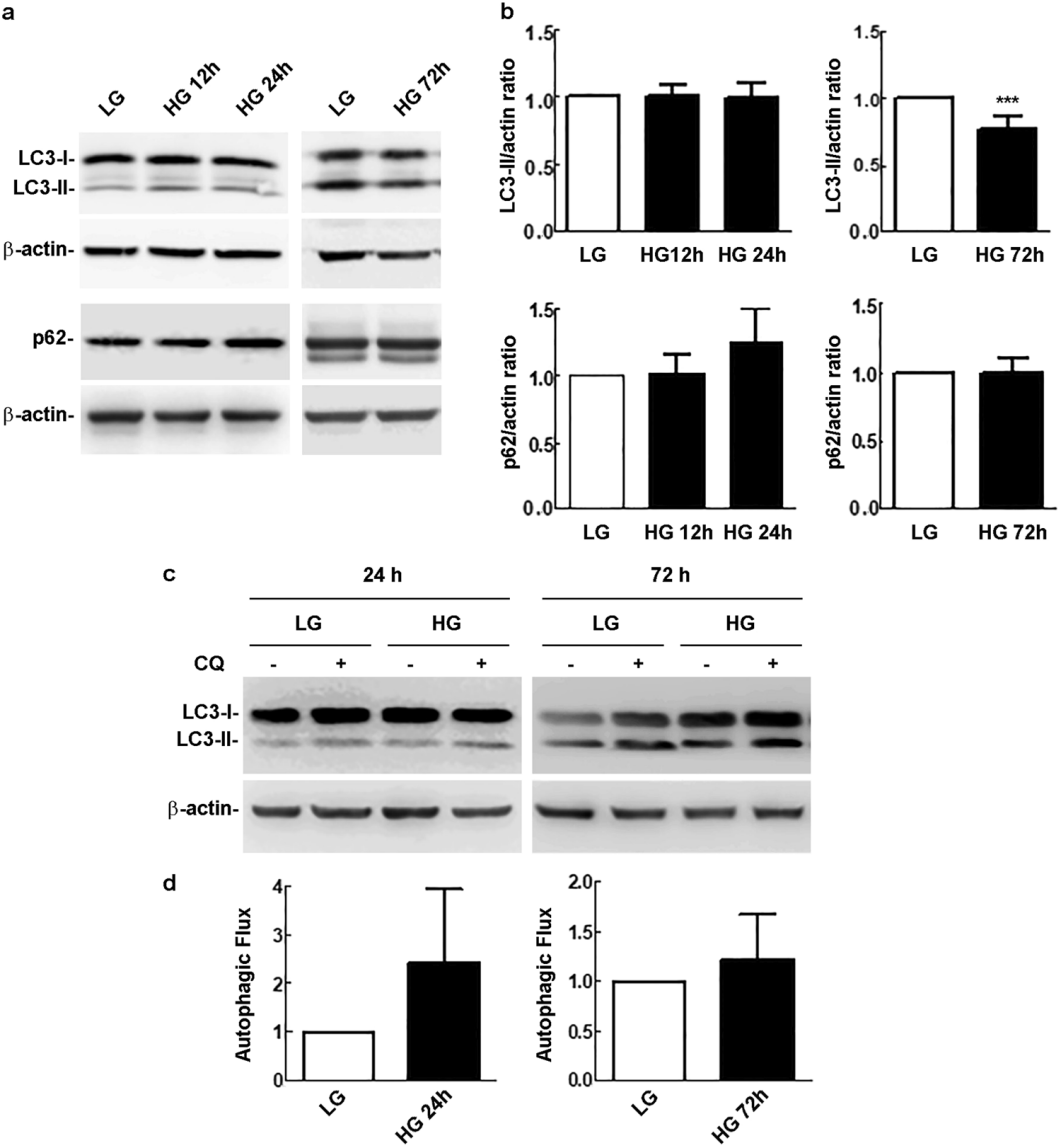
Western blot analysis of autophagy markers in cells exposed to LG or HG conditions for 12–*72* h. **a** LC3-II and p62 levels in cells exposed to LG or HG for 12, 24 and 72 h. **b** Densitometric analysis of the signals obtained in replicates of the experiments shown in **a**. LC3-II/actin and p62/actin ratios of HG-treated samples were normalized to those of control cells maintained in LG, which were arbitrarily set equal to 1. Bars, SEM; ***p < 0.001 vs. LG control cells. **c** LC3-II levels in cells maintained in either LG or HG for 24 and 72 h, in the absence or presence of CQ (20 µM), added 6 h before cell harvesting. **d** Autophagic flux: LC3-II/actin ratios were determined from the densitometric analysis of the signals obtained in replicates of the experiment shown in **c**; the autophagic flux, of cells maintained in either LG or HG, was calculated as the difference between LC3-II/actin levels in the presence and absence of CQ. Bars, SEM

The autophagy markers microtubule-associated protein 1A/1B-light chain 3 (LC3) and p62 were analyzed in rMC-1 cells, at different time points, during 72 h of incubation with 25 mM glucose (HG). A significant LC3-II accumulation, coupled with a reduction of p62 protein levels, was observed in the first 6 h of treatment (Fig. 1a, b), suggesting an early autophagic response of rMC-1 cells to HG. We also measured the difference of LC3-II amount in the presence and absence of the autophagy inhibitor CQ, which represents the amount of LC3-II that is delivered to lysosomes for degradation during the CQ incubation time and is an indirect measure of the autophagic flux (Fig. 1c, d). After 6 h of HG stimulation, the autophagic flux was greater than that of cells maintained under low glucose conditions (LG), confirming a glucose-induced early autophagic response. However, LC3-II and p62 levels returned to values similar to those of untreated control cells in the time interval 12–72 h (Fig. 2a, b). Moreover, the autophagic flux of cells exposed for either 24 or 72 h to HG was not significantly different from that of cells maintained in LG (Fig. 2c, d), confirming that autophagy is not reactivated in Müller cells for up to 3 days of HG exposure.

### Effects of medium hyperosmolarity

One of the consequences of hyperglycemia is osmotic stress, which affects a wide variety of cell types, including retinal cells [17]. Mannitol is commonly used as a control for the effect of hyperosmolarity in cells exposed to HG conditions. We thus incubated rMC-1 cells in a hyperosmolar control medium (5 mM glucose and 20 mM mannitol, MAN) for 2–6 h, and observed an accumulation of both LC3-II and p62 (Fig. 1S), suggesting a mannitol-induced autophagy impairment.

We also analyzed the expression of aquaporin 4 (AQP4) under HG stimulation. AQP4 plays a crucial role in the transport of water across plasma membranes. Indeed, water flux through AQP4 is involved in the rapid volume regulation of retinal Müller cells [18], and AQP4 expression is modulated by hyperosmotic stress in rat astrocytes and human retinal pigment epithelial cells [19, 20]. On the other hand, an increase of AQP4 is considered an early marker of Müller cells activation [21]. Still, under our experimental conditions we observed no significant modifications in AQP4 levels in rMC-1 cells exposed to HG for 2–24 h (Fig. 2S).

### The TRAF2-JNK pathway is not involved in the early activation of autophagy induced by HG

**Fig. 3:**
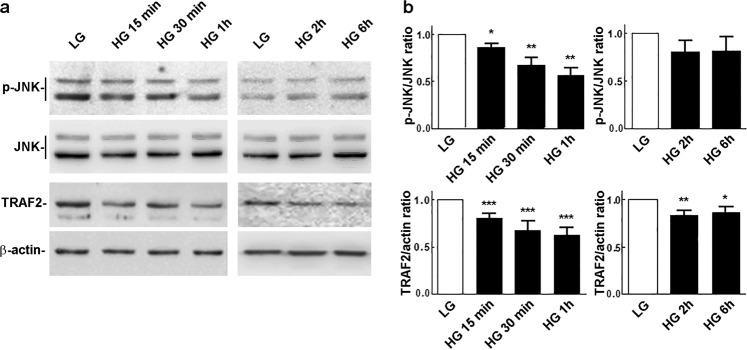
TRAF2-JNK signaling response to HG exposure. **a** Time-course assessment of JNK phospho-activation and TRAF2 protein levels in cells maintained in LG or exposed to HG for the indicated times. **b** pJNK/JNK and TRAF2/actin ratios as determined from the densitometric analysis of the bands obtained in replicates of the experiments shown in **a**. pJNK/JNK and TRAF2/actin values were normalized to those of LG-treated controls, which were arbitrarily set equal to 1. Bars, SEM; *p < 0.05, **p < 0.01 and ***p < 0.001 vs. LG controls

It is well known that ER stress plays an important role in DR [3]. The stress sensors of ER include inositol-requiring enzyme 1 (IRE1), PKR-like ER kinase (PERK) and activating transcription factor 6 (ATF6). While PERK and ATF6 do not play important roles in the activation of autophagy, the IRE1 pathway, is believed to be specifically required for autophagy activation in the early phase of ER stress [22, 23]. In particular, the interaction of IRE1 with tumor necrosis factor receptor-associated factor 2 (TRAF2) results in the activation of apoptosis signal-regulating kinase (ASK-1) and the downstream target c-jun NH2 terminal kinase (JNK). This causes phosphorylation of B-cell lymphoma 2 (Bcl-2) and the disruption of Bcl-2/beclin-1 autophagy repressor complex [24, 25], leading to autophagy activation. Therefore, we analyzed the activation of this pathway within the first 6 h of exposure to HG. Unexpectedly, the activation of JNK was significantly inhibited already 15 min after HG treatment (Fig. 3a, b), and it gradually returned after 2 h of treatment to values similar to those of cells maintained in LG. A sharp decrease of TRAF2 level was also detected at this time and this event correlated with the decline of JNK activation (Fig. 3a, b). These findings demonstrate that the IRE1-TRAF2-JNK pathway is not involved in the early autophagic response of rMC-1 cells to HG exposure.

### HG exposure causes an early increase of ROS and activation of AKT and ERK

**Fig. 4:**
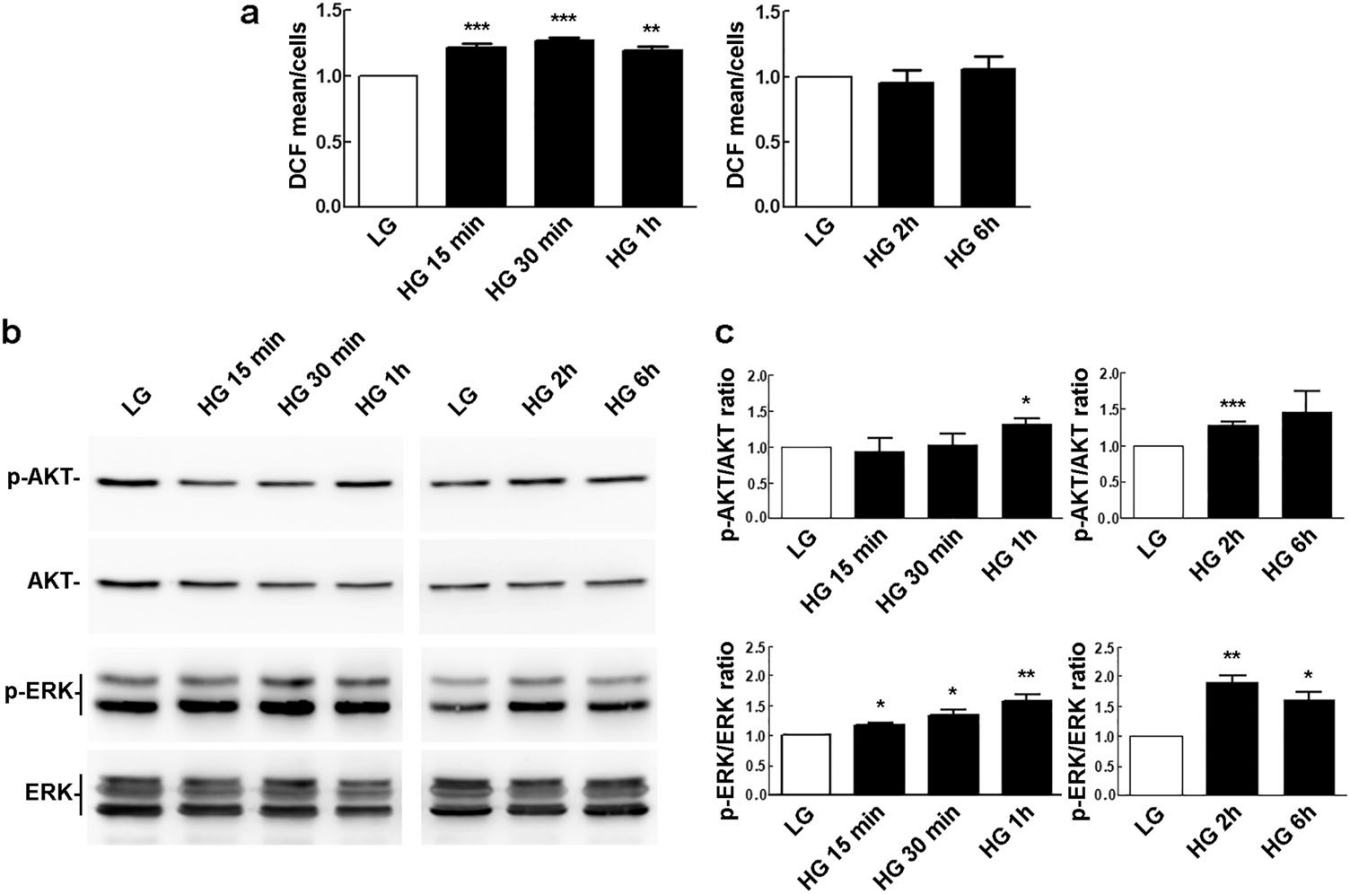
Effect of HG on ROS levels and activation of AKT and ERK. **a** Formation of intracellular ROS, as evaluated using DCFH-DA, in cells maintained in LG or exposed to HG for the indicated times. The mean fluorescence intensity of DCF per cell was normalized to the value obtained with rMC-1 cells maintained in LG. Bars, SEM. **p < 0.01, ***p < 0.001 vs. LG controls. **b** Western blotting assessment of AKT and ERK phospho-activation in cells maintained in LG or exposed to HG for the indicated times. **c** p-AKT/AKT and p-ERK/ERK ratios, as determined from the densitometric analysis of the bands obtained in replicates of the experiments shown in **b**. p-AKT/AKT and p-ERK/ERK values were normalized to those of the respective LG-treated controls, which were arbitrarily set equal to 1. Bars, SEM. *p < 0.05, **p < 0.01, ***p < 0.001 vs. LG controls

HG levels have been related to increased production of ROS [26]. We thus investigated the levels of ROS in rMC-1 cells exposed to HG for up to 6 h. A significant but transient increase in ROS amounts was observed between 15 min to 1 h of HG stimulation (Fig. 4a).

In turn, ROS are known to activate different signaling pathways, including the one mediated by protein kinase B (AKT) [27, 28], and activation of the AKT pathway appears to prevent apoptosis under HG stimulation [29]. Indeed, we observed a transient AKT activation in rMC-1 cells, starting after 1 h of incubation with HG (Fig. 4b, c). Such activation would be expected to hinder autophagy, since phospho-AKT is known to activate mTOR [30], one of the most important inhibitors of autophagy [31]. In fact, in this regard it has been reported that mTOR inhibition restores the activation of autophagy machinery and cargo degradation in rMC-1 cells cultured for 24 h in HG conditions [8].

On the other hand, beside AKT, the extracellular signal regulated kinase (ERK) pathway is also activated by ROS through different mechanisms. For instance, ROS may induce activation of EGF and PDGF receptors as well as an increase of intracellular calcium which, in turn, mediate ERK activation [28].

We thus investigated the involvement of ERK in the early response of rMC-1 cells to HG. Of note, this pathway is known to induce autophagy [32] and plays an important role in glucose metabolism regulation [33–36]. In particular, ERK activation is required to upregulate the expression of the insulin-independent glucose transporter-1 (GLUT1) [37], that is critical for maintaining energy metabolism in Müller glia [38]. In this regard, we recently reported that 96 h exposure of rMC-1 cells to HG triggers a significant increase of phospho-ERK [39]. Here, we show that HG triggers an early and sustained activation of ERK, starting after 15 min of exposure and lasting for the following 6 h (Fig. 4b, c).

### The early autophagy induced by HG requires ERK activity

**Fig. 5:**
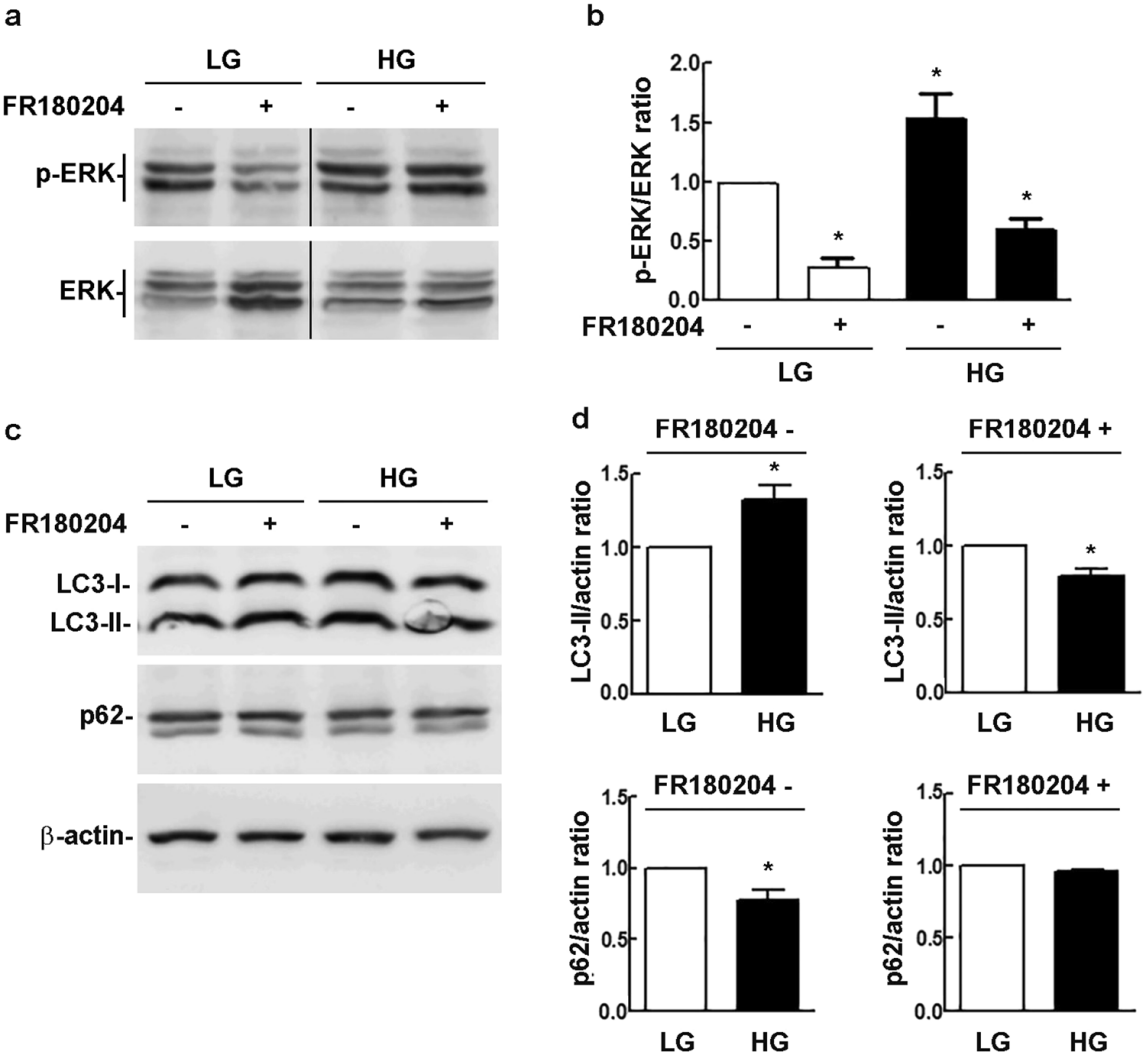
Effect of ERK inhibition on HG-induced autophagy. Representative immunoblots showing **a** p-ERK, **c** LC3-II and p62 levels, in rMC-1 cells exposed to HG for 6 h, in the absence or presence of the ERK inhibitor FR180204, added 2 h before cell harvesting. **b** p-ERK/ERK and **d** LC3-II/actin and p62/actin ratios as determined from the densitometric analysis of the bands obtained in replicates of the experiments shown in **a** and **c**. The p-ERK/ERK, LC3-II/actin and p62/actin values were normalized to those of the respective LG-treated controls, which were arbitrarily set equal to 1. Bars, SEM. *p < 0.05 vs. LG controls

Next, in order to provide evidence for the involvement of ERK in the early activation of autophagy, we evaluated the effect of the ERK inhibitor FR180204 on the induction of autophagy in rMC-1 cells exposed to HG for 6 h (Fig. 5). FR180204 reverted the effect of HG treatment reducing both the increase of LC3-II and the decrease of p62 (Fig. 5c, d), thus confirming that ERK activity is required to trigger early autophagy upon HG exposure.

### Early autophagy enhances viability of rMC-1 cells in HG

**Fig. 6:**
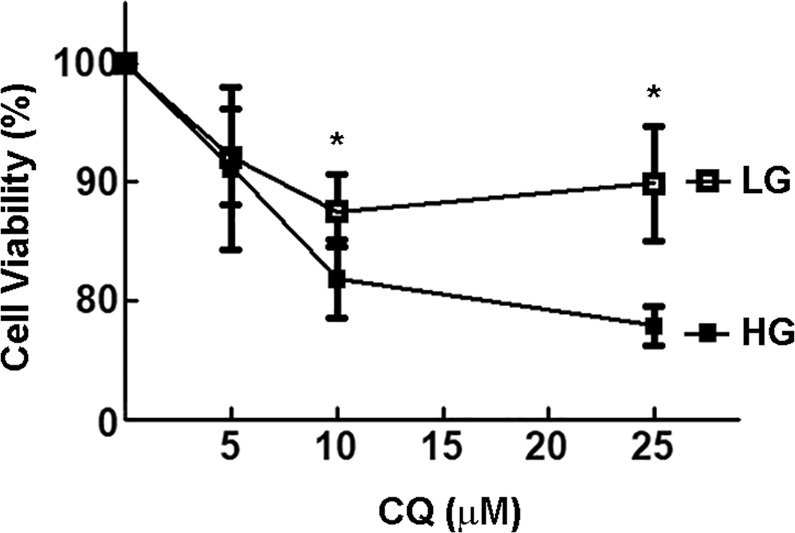
Effect of autophagy inhibition on cell viability. rMC1 were maintained in either LG or HG for 24 h in the absence or presence of increasing concentrations of CQ (1–25 µM). Cell viability was then evaluated by SRB assay. *p < 0.05 vs. LG control cultures

Finally, we analyzed the impact of autophagy inhibition on cell viability after 24 h exposure to either LG or HG (Fig. 6). As far as cell viability is concerned, Vellanki et al. showed that a 24 h treatment with HG significantly increased the viability of rMC-1 and of human Müller cell line MIO-M1, compared to cells maintained in normal glucose (5 mM) [40]. This evidence was also confirmed by our recent findings showing perfectly viable rMC-1 cells, after 96 h of constant exposition to HG [39]. However, a 24 h treatment with 25 µM CQ reduced cell viability of ~10% in LG conditions, while the effect was almost two times higher in HG conditions. These results demonstrate that autophagy inhibition renders rMC-1 cells more sensitive to HG exposure suggesting that the early autophagy induction by HG is part of a survival mechanism aimed at coping with stress.

## Discussion

A number of studies have investigated the effect of HG on autophagy in the rat retinal Muller cell line rMC-1. According to the reported results, autophagy is inhibited in the first 24–48 h, and activated at later stages, i.e., after about 72 h to 5 days of cells exposure to HG [8, 10–12]. Of note, this later autophagy activation appears related to mitochondrial dysfunctions caused by sustained HG exposure [11, 12].

With these premises, we investigated rMC-1 Müller cells autophagic activity in the first hours of HG exposure. Indeed, the early response to HG in terms of autophagy regulation had not been investigated so far to our knowledge. The results obtained and reported here demonstrate that HG actually induces an early autophagic response that peaks in the first 6 h of exposure, and that is followed by a decline in the time frame between 12 and 72 h from stimulation. These findings, coupled with those reported above, indicate that a biphasic induction of autophagy may actually occur in retinal Müller cells, in stress conditions associated with a HG environment.

We further analyzed the contribution of different factors to this early autophagy response. The effect of medium hyperosmolarity was analyzed by replacing glucose with mannitol. Of note, the effect of hyperosmolarity on autophagy is a controversial issue, since both activation [41–43] and inhibition of autophagy [44, 45] have been reported to occur after osmotic stress. Under our experimental conditions, the mannitol-induced increase in medium osmolarity inhibited autophagy at early time points. These results suggest that HG exposure could affect autophagy through multiple mechanisms including an hyperosmolarity-mediated inhibition, counteracted by other signaling events that in turn induce early autophagy activation.

Next, we investigated the possible activation of pathways known to play crucial roles in the stress response to HG. HG challenge did not increase AQP4, a marker of retinal Müller cells activation, nor it induced activation of JNK, a kinase that mediates cellular responses to oxidative stress [46]. In addition, the activation of the IRE1–JNK pathway is a key event linking ER stress to autophagy induction [22, 23]. However, our results demonstrate that in rMC-1 cells the early autophagic response to HG is not activated by this pathway. Overall, these findings indicate that a few hours exposure to HG does not induce stress markers in rMC-1 cells.

Actually, we observed a transient increase of ROS, starting after few minutes of HG exposure. However, beside their effect on oxidative stress and JNK activation, low levels of ROS are involved in the regulation of physiological processes via different signaling pathways [47]. Among these, is the ERK pathway, which primarily mediates proliferative and cell survival responses [28, 48], and has been found to play a role in autophagy activation [32]. Indeed, we observed an early and sustained activation of ERK in HG-treated rMC-1 cells. This is in accordance with previous findings showing a direct relationship between ERK activation and glucose availability [32]. Further, we show that the early autophagy induced by HG is impaired upon ERK inhibition, confirming that ERK is required for the autophagic response of rMC-1 cells in the first hours of HG stimulation.

After 1 h of HG exposure, we also observed activation of AKT, a known autophagy inhibitor. Still, the AKT activation does not necessarily result in autophagy inhibition, the actual outcome depending on the balance between the activity of different signaling pathways, including the one mediated by ERK. In this respect, significant cross-talks have been found to occur between ERK and AKT pathways [49]. Moreover, acute activation of ERK has been reported to inhibit mTOR, resulting in cytoprotective autophagy [50].

An important finding in this scenario is that cells exposed to HG had a lower viability upon autophagy inhibition by CQ, as compared to those maintained in LG. Therefore, the HG-induced early autophagy appears to be required to prevent cell damage.

In conclusion, a short-term HG challenge triggers in rMC-1 cells a process improving the ability to cope with stressful conditions, which involves ERK and autophagy. However, this early autophagy activation is followed by a decline at later time points, in agreement with previous reports [8]. Further, other studies have shown that autophagy is induced again under chronic exposure to HG in association with the onset of mitochondrial dysfunctions [11, 12].

Overall, these results indicate that HG exposure may induce in rMC-1 cells a non-linear response characterized by oscillations in autophagic activity. In this respect, it has been recently reported that upon various types of stress stimuli, including serum and glucose starvation, glutamine deprivation, etc., different cells show autophagy oscillations. These fluctuations are characterized by an early autophagic peak, after about 6 h from stimulation, and a later peak coupled with an intermittent drop in autophagic activity [51]. The first peak is interpreted as an attempt to cope with the stressful factor, while the second is regarded as a result of prolonged stress conditions possibly leading to cell death [51].

We previously reported that short-term intermittent exposure to HG triggers in rMC-1 cells an increase of markers of reactive gliosis [39]. Glucose variability is an important factor in the development of diabetic complications, including DR [52]. While intermittent exposure to HG has been reported to induce cytoprotective autophagy in retinal pigmented epithelial cells [53], future investigations will be needed to define the involvement of autophagy in the susceptibility of rMC1 cells to glucose fluctuations.

## Supplementary information

Fig. 1S

Fig. 2S

Supplementary Figure Legends

## Abbreviations

DR: diabetic retinopathy
rMC-1: retinal Müller cells
LG: low glucose
HG: high glucose
ER: endoplasmic reticulum
CQ: chloroquine
AQP: aquaporin
LC3: microtubule-associated protein 1A/1B-light chain 3
p62: sequestosome 1
ERK: extracellular signal regulated kinase
AKT: protein kinase B
JNK: c-Jun N-terminal kinase
TRAF2: TNF receptor-associated factor 2
IRE1: inositol-requiring enzyme 1
PERK: PKR-like ER kinase
ATF6: activating transcription factor 6
GLUT1: glucose transporter-1
SRB: sulforhodamine B
ROS: reactive oxygen species
DCFH-DA: dichlorofluorescein diacetate
