# Supplementary figures and images for: High glucose induces an early and transient cytoprotective autophagy in retinal Müller cells
Source: Endocrine. 2022 May 25;77(2):221–30. doi: 10.1007/s12020-022-03079-8 (PMC9325829; doi:10.1007/s12020-022-03079-8)

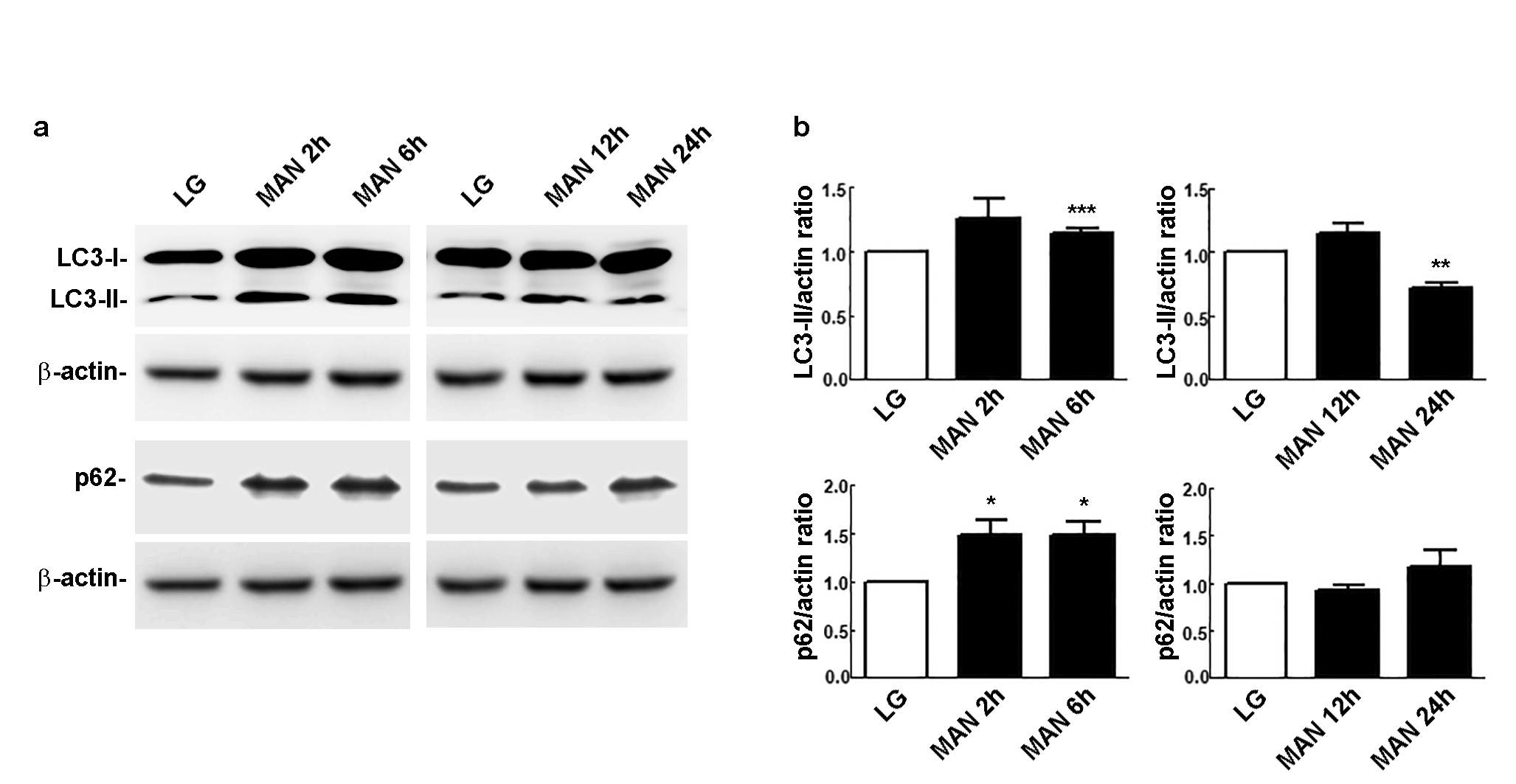

Supplement: Supplementary file 1 — Fig. 1S [file 12020_2022_3079_MOESM1_ESM.jpg]

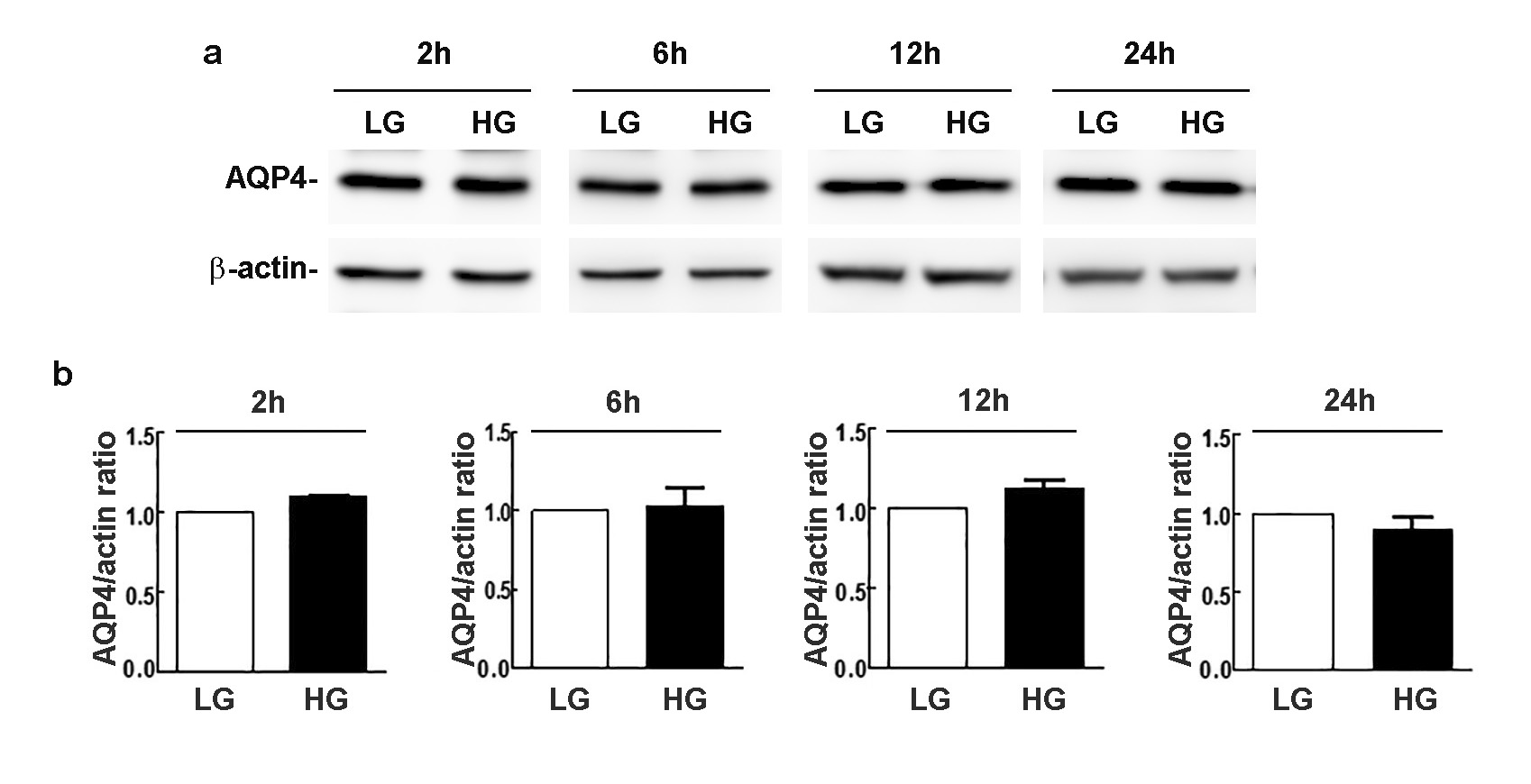

Supplement: Supplementary file 2 — Fig. 2S [file 12020_2022_3079_MOESM2_ESM.jpg]
