# Supplementary material for: High glucose induces an early and transient cytoprotective autophagy in retinal Müller cells
Source: Endocrine. 2022 May 25;77(2):221–30. doi: 10.1007/s12020-022-03079-8 (PMC9325829; doi:10.1007/s12020-022-03079-8)
Supplement: Supplementary file 3 — Supplementary Figure Legends [file 12020_2022_3079_MOESM3_ESM.docx]

**Fig. 1S** Western blot analysis of autophagy markers in LG or mannitol-treated (MAN) rMC-1 cells. (a) Time-course assessment of LC3-II and p62 protein levels in rMC-1 cells exposed to a medium containing 5 mM glucose and 20 mM mannitol for 2, 6, 12, and 24 hours. (b) Densitometric analysis of the signals obtained in replicates of the experiments shown in a. LC3-II/actin and p62/actin levels of MAN samples were normalized to those of control cells maintained in LG, which were arbitrarily set equal to 1. Bars, SEM; *p < 0.05, **p < 0.01 and ***p < 0.001 vs LG controls.

**Fig. 2S** Effect of HG on AQP4 levels. (a) Representative immunoblots showing AQP4 levels in rMC-1 cells maintained in LG or exposed to HG for the indicated times. (b) AQP4/actin ratios as determined from the densitometric analysis of the bands obtained in replicates of the experiments shown in a. AQP4/actin values were normalized to those of controls maintained in LG, which were arbitrarily set equal to 1. Bars, SEM.
